# Pregnancy, Postpartum, and Parenting Engagement Resource Center: A national resource for researchers, community organizations, and parents with substance use disorders

**DOI:** 10.1016/j.dadr.2026.100470

**Published:** 2026-08-18

**Authors:** Camille C. Cioffi, Maya Casper, Leslie D. Leve, David S. DeGarmo

**Affiliations:** University of Oregon, Prevention Science Institute, 1600 Millrace Dr, Eugene, OR 97403, United States

**Keywords:** Parenting, Substance use disorder, Postpartum, Community-engaged research, Recovery

## 1 Why parent-centered SUD research matters

Approximately 40–60% of people with substance use disorders (SUD) in treatment are parents (Canfield et al., 2021; T. J. McMahon et al., 2005a) and nearly 19 million children under age 18 lived with one or more parents with an SUD (McCabe et al., 2025). Most people with SUD have also had pregnancy experiences (Cioffi and Seeley, 2021). Drug overdose is now a leading cause of death during pregnancy and postpartum (Azad et al., 2026), and substance use-related deaths are the second leading cause of death during the peripartum period, after obstetric complications (Goldman-Mellor and Margerison, 2019, Margerison et al., 2022). Among pregnant people, substance use is associated with a fourfold increase in the odds of death during the birthing hospitalization (Addis et al., 2001, American College of Obstetricians and Gynecologists, 2011, Krawczyk et al., 2020, Sanjanwala and Harper, 2019). Together, these findings underscore the need to better support treatment engagement and recovery among people with SUD who are pregnant, postpartum, or parenting. Parents with SUD are experts in their own experiences, and their perspectives should be incorporated and emphasized into the questions, methods, and services intended to support them. Researchers may lack the infrastructure needed to meaningfully engage parents with lived experience or reach them for participation in research. The purpose of this commentary is to introduce the Pregnancy, Postpartum, and Parenting Engagement Resource Center (PERC) and describe the free resources available to U.S. researchers to advance parent-centered SUD and SUD services research.

## 2 A National research resource: the Pregnancy, Postpartum, and Parenting Engagement Resource Center (PERC)

PERC was developed to advance a parent-centric research agenda focused on community-drive research that improves treatment engagement and outcomes for parents with SUD. PERC builds directly on prior infrastructure and lessons learned through sustained engagement with parents with SUD. In 2019, our P50 NIDA Center of Excellence, the Center on Parenting and Opioids (CPO), was established as a national resource to improve parenting practices among parents with opioid use disorder and other SUD. Although the CPO’s research projects were child-centric, meaningful engagement with this population underscored the need to better understand and address the unique experiences and needs of parents with SUD. In response, CPO launched the *Data Collective* in 2022 as a national recruitment and engagement resource for research with parents with SUD. In 2023, CPO established a *Lived Experience Community Board (LECB),* laying the foundation for PERC.

PERC extends this infrastructure by providing researchers with resources to more readily conduct parent-centered SUD research. These resources are available to researchers and trainees across the United States at no cost.

### 2.1 PERC resources for researchers

Researchers can use PERC’s free resources to support parent-centered SUD research at multiple stages of the research process. PERC can connect researchers with parents with lived experience through the *LECB*, provide consultation on research questions, study design, recruitment and retention strategies, measures, interpretation, and dissemination, and support recruitment through the national *Data Collective* of parents who have agreed to be contacted about research opportunities. PERC also provides training and guidance to help researchers engage parents with SUD in ways that are meaningful, ethical, and non-stigmatizing. Table 1 provides an overview of PERC resources, what each resource offers researchers, and how researchers can use them throughout the research process. Together, these resources provide researchers with established infrastructure for incorporating lived experience and reaching potential participants without having to build these partnerships and recruitment systems independently.

**Table 1:** PERC resources available to researchers.

PERC Resource	What PERC Offers Researchers	How to access
Lived Experience Community Board (LECB)	*Consultation with parents with lived experience of SUD* to inform research questions, study design, measures, recruitment and retention strategies, interpretation of findings, and dissemination.	Submit a request at: https://perc.uoregon.edu/resources-for-researchers/
Special Emphasis Panels (SEPs)	*Focused consultation* with parents whose lived experiences are relevant to specific populations or topics such as rural contexts, fathering, pregnancy, parenting children of different ages, child welfare involvement, and pregnancy or infant loss.	Indicate special emphasis panel in LECB interest form
Data Collective	*Access to national cohort of recruitable parents* with histories of substance use who have agreed to be contacted about research opportunities.	Submit a request at: https://perc.uoregon.edu/resources-for-researchers/
Researcher Training	*Free asynchronous training and practical resources* for working with parents with SUD to strengthen ethical, meaningful, and non-stigmatizing community engagement, recruitment, and research communication.	Provided after a request is approved
Internship Partnerships	*Training and supervision infrastructure for students* conducting community-based research which supports the Data Collective as a national resource alongside training opportunities for next generation.	Contact the study team to discuss site institutional specific partnerships

### 2.2 Lived Experience Community Board (LECB)

#### 2.2.1 Partnering with the LECB

Researchers can partner with PERC’s *LECB* to incorporate the perspectives and priorities of parents with lived experience of SUD throughout the research process. The *LECB* includes 16 parents with lived experience of SUD who are prepared to provide meaningful input on research related to pregnancy, postpartum, parenting, and substance use. Researchers can engage the *LECB* during the development of grant applications and research questions, study design, selection and development of measures, recruitment and retention planning, interpretation of findings, and development of dissemination materials. Thus, the *LECB* provides researchers with an established mechanism for obtaining parent-informed feedback without first having to develop a community advisory infrastructure independently. Researchers interested in collaborating the *LECB* submit a brief request form (https://perc.uoregon.edu/resources-for-researchers/).

*LECB* members are prepared for research consultation through a structured training series covering the purpose and funding of research; foundational concepts such as how research questions, hypotheses, and testing are developed; and key principles of research ethics. The trainings also offered information on how research priorities are and can be set, how studies are designed, who is included in research, and how findings are interpreted and disseminated. Training videos were developed after formative input from the *LECB* on their prior training experiences and training preferences. Training videos are hosted on the PERC website for use by other community boards (https://blogs.uoregon.edu/perc/training/).

Researchers seeking perspectives related to specific populations or experiences can also request consultation with one of PERC’s Special Emphasis Panels (SEPs). Currently available SEPs provide focused lived-experience perspective related to rural contexts; fathering; pregnancy; parenting young children; parenting adolescents; families involved with child welfare systems; and families who have experienced loss, including pregnancy or infant loss. Researchers who wish to engage *LECB* first submit a brief interest form describing their project and consultation needs. Researchers whose projects align with PERC’s mission complete a brief researcher training, and access an accompanying resource guide, and available consultation dates.

#### 2.2.2 What researchers can expect from an LECB consultation

*LECB* consultations are structured as bi-directional discussions with researchers regarding grant applications, existing research projects, or emerging research results. Researchers bring specific questions, materials, or challenges to the *LECB* and receive actionable feedback from parents with lived experience. Depending on the stage of the research, consultation may include feedback on the relevance of research questions and outcomes, study procedures and measures, recruitment and retention strategies, participant-facing language and materials, interpretation of emerging findings, and approaches for communicating results to parents with SUD and the organizations that serve them. Researchers may engage with the *LECB* during a single stage of a project or return at multiple points throughout the research process.

#### 2.2.3 Preparing researchers for LECB engagement

PERC provides researchers with brief, asynchronous training and practical resources to support ethical, effective, and non-stigmatizing engagement with parents with SUD. The training introduces PERC’s community-engaged approach and provides guidance that researchers can apply both during LECB consultation and in their broader research (https://blogs.uoregon.edu/perc/resources-for-researchers/). Topics include strategies for meaningful engagement with people with lived experience, best practices for recruitment and retention, and the use of non-stigmatizing language and imagery in research materials and dissemination (Condensed Language Style Guide, 2020; Lipsett et al., 2023). Following the training, researchers complete a brief knowledge assessment and, upon satisfactory completion, sign an agreement outlining expectations for engaging with PERC resources. For researchers engaging with the *LECB*, these expectations include preparing accessible materials, identifying specific goals for consultation, establishing the anticipated level of engagement, and returning study findings to *LECB* members. Researchers also receive a best-practice guide and engagement checklist to help translate these principles into practice throughout their project.

**Fig. 1:**
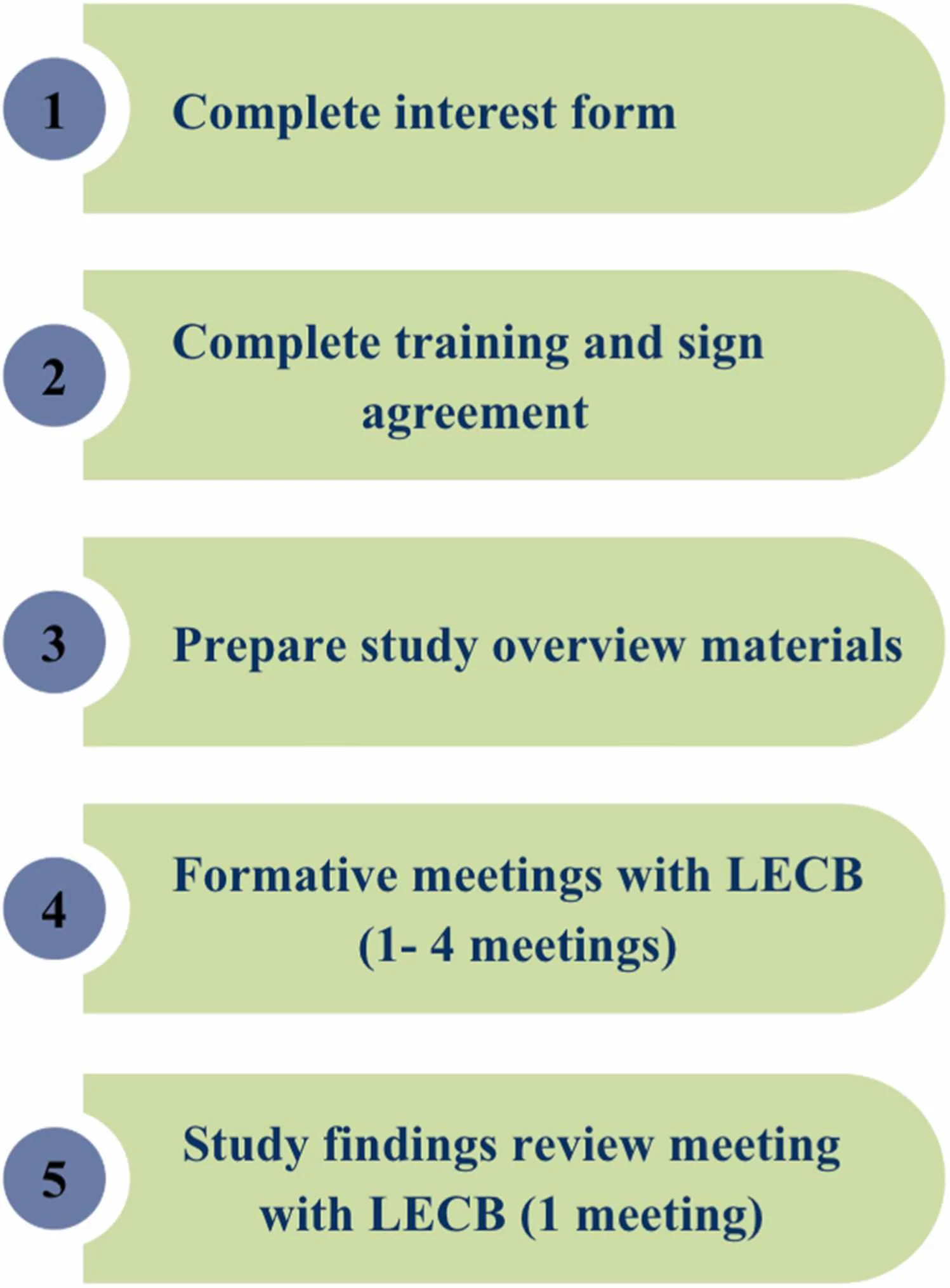
Process Overview for LECB Engagement.

PERC staff help researchers ensure that information provided to *LECB* is concise, accessible, non-stigmatizing, and sufficient for members to provide meaningful feedback. Researchers are provided an accompanying resource guide and available consultation dates. Researchers then meet with the PERC team to identify goals for the consultation and review proposed materials. This preparation is intended to maximize time for discussion and actionable input during the consultation rather than extended presentation of study background. An overview of this process is provided in Fig. 1, and a sample meeting structure is provided in Table 2.

**Table 2:** Example LECB meeting series.

**Meeting**	**Timeline**	**Focus**	**Example Questions/Activities**
1	Month 1, Week 1	Background information Gather formative feedback	Share one-page overview in advance What do you think is important to learn from a study on [topic]? What do you think we will learn from participants?
2	Month 1, Week 3	Gather feedback on intervention protocol	What would you think of this intervention if you were participating? Who do you think will most benefit from this intervention? What could we do to make the intervention more useful?
3	Month 2, Week 1	Gather feedback on data collection instruments	Offer survey for participants to give feedback on groups of items either as a group or independently
4	Month 2, Week 3	Gather feedback on engagement protocols	What are things we can do to help participants feel comfortable participating? What might motivate individuals to participate? What might get in the way?
5	Month 8	Return study findings	Share one-page overview in advance What did you find interesting about the findings? What do you think other parents with SUD will be most interested in knowing? How should we share the information with them?

Researchers and *LECB* members are invited to provide feedback about their collaboration, including the extent to which parents were engaged in different stages of the research and how their input influenced research decisions. PERC uses this information to continuously improve its consultation model and provide researchers with guidance for strengthening future community-engaged work. Through this combination of training, structured consultation, and ongoing feedback, PERC provides researchers with practical infrastructure for developing meaningful partnerships with parents with lived experience of SUD.

Following collaboration with the *LECB*, when feasible, researchers are encouraged to engage *LECB* members as compensated members of their research teams (e.g., grant writing, information synthesis, data collection, data interpretation, creation of dissemination materials).

### 2.3 *Data Collective*

#### 2.3.1 Connecting researchers with participants through the *Data Collective*

Researchers can use PERC’s *Data Collective* to connect with potential research participants who have experienced SUD in the context of pregnancy, postpartum, and parenting. The *Data Collective* is a national research cohort of individuals with a history of substance use who are parenting, have a recent pregnancy history, or plan to become parents within the next five years. Eligible participants report active use of substances other than tobacco or cannabis within the past year, or have a lifetime history of treatment for a SUD (they may additionally report tobacco and/or cannabis use, but these cannot be the sole substances they report). Individuals voluntarily enroll in the *Data Collective* and consent to be contacted about future research opportunities related to pregnancy, postpartum, parenting, and SUD. Participants retain the choice of whether to respond to or participate in each research opportunity they receive and receive incentives for their participation.

The *Data Collective* provides researchers with access to an established recruitment infrastructure that reaches parents across urban, mid-sized, and rural communities. Between December 2022 and August 10, 2026, 810 participants enrolled in the *Data Collective*. Participants have a mean age of 37 years; 8.9% identify as American Indian or Alaska Native, 7.3% as African American or Black, 17% as Latine/x, and 76% as White; 62% identify as women. Sixty-four percent reported substance use other than tobacco in the past three months, with high lifetime exposure to fentanyl (54%) and methamphetamine (80%). These characteristics allow researchers to assess whether the *Data Collective* may be an appropriate recruitment resource for their study population.

Through 2028, PERC will be maintaining ongoing engagement with *Data Collective* participants rather than contacting them only when recruitment opportunities arise. While enrolled, participants complete follow-up surveys every six months to update eligibility for future studies and to contribute longitudinal data on parenting behaviors, substance use, mental health, and contextual factors. *Data Collective* participants also receive monthly text messages with resources such as overdose prevention information, parenting resources, and ideas for fun family activities. This continued engagement helps PERC maintain an up-to-date cohort that can be matched with relevant research opportunities.

Community-based organizations that partner with PERC receive quarterly reports summarizing aggregate information about participants recruited through their organization, including demographics, substance use, and satisfaction with services. Participants can also provide feedback about organizational strengths and opportunities for improving services, providing community partners with information that can support service improvement.

#### 2.3.2 Recruitment support through the *Data Collective*

PERC provides researchers with recruitment support through the *Data Collective* at no cost, through 2028. Researchers interested in using the *Data Collective* submit a brief request form describing their study, eligibility criteria, and recruitment needs (https://perc.uoregon.edu/resources-for-researchers/). Researchers also provide ethics board approval, the study protocol and consent form, and approved text and email recruitment materials. PERC works with researchers to ensure that recruitment messages are brief, clearly describe available incentives, and provide potential participants with a clear way to express interest, such as a study website or telephone number.

Once a request is approved, PERC manages outreach to potentially eligible *Data Collective* participants on behalf of the external research team. Using the researcher’s approved recruitment language, PERC sends up to three text messages and emails to potentially eligible participants. Researchers may also request additional telephone outreach, in which case the PERC research team makes one phone call to potentially eligible participants to share the research opportunity and encourage interested individuals to connect with the study team. This approach allows researchers to recruit from an established national cohort while PERC manages participant identification and initial outreach.

#### 2.3.3 Supporting successful *Data Collectiv*e partnerships

PERC provides researchers with brief training and guidance to support effective use of the *Data Collective.* Researchers complete a condensed version of the asynchronous training and knowledge assessment described for *LECB* engagement, which introduces PERC, the *Data Collective*, and best practices for engaging and recruiting parents with SUD. Unlike *LECB* consultation, use of the *Data Collective* does not require a preparatory meeting with the PERC team.

PERC also works with researchers to understand the reach of *Data Collective* recruitment efforts and continuously improve the resource. Researchers are encouraged to ask participants how they learned about the study or, when appropriate, obtain consent to notify PERC when a *Data Collective* participant enrolls. This information helps PERC understand which outreach strategies successfully connect participants with research opportunities and strengthen future recruitment efforts. Partnership with the *Data Collective* can also be reciprocal. Researchers who recruit eligible parents through other pathways may refer interested individuals to the *Data Collective*, allowing participants to learn about additional research opportunities and helping expand the national resource available to the broader research community.

### 2.4 Opportunities for researchers and trainees to partner with PERC

PERC also provides opportunities for researchers to establish *Data Collective* recruitment and student training partnerships in their local communities. PERC offers a two-term/semester internship training program that prepares students to engage parents with SUD in community-based research. The first term focuses on understanding SUD in the context of pregnancy and parenting, recruitment best practices, and use of the *Data Collective* database for participant outreach and tracking. During the second term, interns gain field research experience at a community placement site. Researchers interested in developing a *Data Collective* recruitment site in their local community can partner with PERC to establish an internship placement. PERC coordinates and oversees the internship program, providing a structured training curriculum, standardized recruitment procedures and materials, *Data Collective* infrastructure, and ongoing supervision of student interns. PERC also works with local researchers to identify and establish relationships with appropriate community placement sites.

Once established, PERC manages intern training, preparation for field-based recruitment, ongoing supervision, and *Data Collective* recruitment and tracking procedures. This structure allows researchers to expand *Data Collective* recruitment and build community partnerships in their local area while PERC provides the infrastructure and oversight needed to implement and sustain the internship program. Contact the study at parentingcenter.uoregon.edu to coordinate an internship placement.

## 3 Conclusion

PERC provides a free national resource for researchers seeking to conduct meaningful, parent-centered research related to pregnancy, postpartum, parenting, and SUD. Through the *Data Collective*, *LECB*, researcher training, and other partnership opportunities, PERC provides established infrastructure to help researchers connect with potential participants, incorporate lived experience throughout the research process, and conduct research that is responsive to the priorities and needs of parents with SUD. These resources are available to researchers and trainees across the United States at no cost, through at least 2028. Funding thereafter is contingent upon securing additional sources of support for this resource.

Researchers are encouraged to engage with PERC at any stage of the research process, from developing research questions and grant applications to recruiting participants, interpreting findings, and developing dissemination materials. By making community-engagement and recruitment infrastructure broadly accessible, PERC aims to reduce barriers to conducting parent-centered research and support research that can more effectively inform practice and improve outcomes for parents and families.

## CRediT authorship contribution statement

**Maya Casper:** Project administration, Writing – original draft, Writing – review & editing. **David S. DeGarmo:** Methodology. **Leslie D. Leve:** Funding acquisition, Writing – original draft, Writing – review & editing. **Camille C. Cioffi:** Conceptualization, Funding acquisition, Supervision, Writing – original draft, Writing – review & editing.

## Author disclosures (document)

You can use the Author Disclosures page to add any additional information about the manuscript.

## Ethical approvals

Protocol Number: STUDY00000617, University of Oregon Institutional Review Board.

## Ethics declaration

Written informed consent to take part in the study and to publish the article has been obtained from all participants or their legal representatives. The privacy rights of participants have been observed.

This study was performed in compliance with relevant laws, regulatory frameworks and guidelines where the research took place. This study was approved by the University of Oregon Institutional Review Board. (Approval No. STUDY00000617)

## Funding

The activities described in this article were supported by Grants R24DA061209 & P50DA048756 from the 10.13039/100000026National Institute on Drug Abuse, 10.13039/100000002National Institutes of Health (NIH). The content is solely the responsibility of the authors and does not necessarily represent the official views of the NIH.

## Role of the funding source

The funder had no role in study design, data collection and analysis, decision to publish, or preparation of the manuscript.

## Declaration of Competing Interest

The authors declare that they have no known competing financial interests or personal relationships that could have appeared to influence the work reported in this paper

## Data Availability

The PERC Data Collective data are available from the Inter-university Consortium for Political and Social Research (ICPSR) at https://doi.org/10.3886/E229562V1.
